# Silencing UBQLN2 Enhances the Radiosensitivity of Esophageal Squamous Cell Carcinoma (ESCC) via Activating p38 MAPK

**DOI:** 10.1155/2023/2339732

**Published:** 2023-01-05

**Authors:** Jia-Lin Wang, Xiao-Ying Mu, Rong Ma, Xue-Hong Bai, Zhi-Jun Zhao, Yan-Yang Wang

**Affiliations:** ^1^Department of Radiation Oncology, General Hospital of Ningxia Medical University, Yinchuan 750004, Ningxia, China; ^2^Graduate School, Ningxia Medical University, Yinchuan 750004, Ningxia, China; ^3^Cancer Institute, Ningxia Medical University, Yinchuan 750004, Ningxia, China; ^4^Department of Laboratory Medicine, General Hospital of Ningxia Medical University, Yinchuan 750004, Ningxia, China; ^5^Institute of Medical Sciences, General Hospital of Ningxia Medical University, Yinchuan 750004, Ningxia, China

## Abstract

**Background:**

Ubiquilin 2 (UBQLN2) is an adaptor of ubiquitinated proteins and the proteasome. The potential role of UBQLN2 in carcinogenesis has been demonstrated. However, its role in modulating the radiosensitivity of cancer is not clear. Here, we explored the radiosensitizing effect of silencing UBQLN2 on esophageal squamous cell carcinoma (ESCC) and its mechanisms.

**Methods:**

We analyzed the prognostic role of UBQLN2 in the ESCC patient cohort from the Cancer Genomic Atlas (TCGA) database and our hospital. We also conducted a series of experiments in vivo and in vitro to investigate the effect of silencing UBQLN2 on ESCC radiosensitivity and its mechanisms.

**Results:**

UBQLN2 is highly expressed in ESCC tissues and positively correlated with poor overall survival (OS). The knockdown of UBQLN2 dramatically increased the radiosensitivity of ESCC cells. Mechanically, UBQLN2 suppression substantially upregulated p38 mitogen-activated protein kinases (MAPK). The p38 MAPK inhibitor SB203580 could reverse the radiation-enhancing effect induced by UBQLN2 knockdown. The direct interaction between UBQLN2 and p38 MAPK was confirmed by co-immunoprecipitation (CO-IP) assay. Furthermore, silencing UBQLN2 also inhibited the expression of phosphorylated DNA-dependent protein kinase catalytic subunit (p-DNA-PKcs) after irradiation. Finally, the xenografted tumor experiment confirmed the radiosensitizing effect of silencing UBQLN2 on ESCC in vivo.

**Conclusion:**

Our results suggest that silencing UBQLN2 enhances the radiosensitivity of ESCC by activating p38 MAPK, and UBQLN2 may be a potential target to enhance the radiosensitivity of ESCC.

## 1. Background

Esophageal cancer is one of the most common malignant tumors and a leading cause of cancer-related deaths in the world. In China, squamous cell carcinoma constitutes the majority of esophageal cancer patients, while esophageal adenocarcinoma is the prevalent type in North America and Western Europe [1, 2]. Radiotherapy is the first choice for locally advanced-stage esophageal squamous cell carcinoma (ESCC) patients or early-stage ESCC patients with high risk. Although progress has been made in radiotherapy techniques for esophageal cancer, the outcome of patients with ESCC is bleak [3]. One of the approaches to improve the efficacy of radiotherapy is to enhance the radiosensitivity of ESCC [4].

Ubiquilin 2 (UBQLN2) is one of four homologous UBQLN proteins expressed in humans. It contains a ubiquitin-like domain (UBL) at the *N*-terminal and a ubiquitin-associated domain (UBA) at the C-terminal. The function of the UBA domain is to bind the ubiquitin part, while the UBL domain binds to the S5a subunit in the proteasome. Therefore, UBQLN2 is an important regulator of protein homeostasis and acts as a shuttle factor to transport misfolded proteins to the proteasome for degradation [5–7]. It is reported that UBQLN2 is associated with a variety of neurological diseases, including polyglutamine disease, Alzheimer's disease, and amyotrophic lateral sclerosis (ALS) [8]. In addition to neurological diseases, UBQLN2 also plays an important role in malignant tumors [9–15]. In our previous work, we found that UBQLN2 had a potential role in the pathogenesis of ESCC [16]. However, there is limited information about the role of UBQLN2 in regulating the radiosensitivity of ESCC.

Here, we studied the molecular role of UBQLN2 in modulating the radiosensitivity of ESCC. Our results revealed that the downregulation of UBQLN2 significantly enhanced the radiosensitivity of ESCC. In addition, we also found that p38 mitogen-activated protein kinases (MAPK) were a downstream regulator of UBQLN2 and participated in the mediation of radiosensitivity. The inhibition of p38 MAPK could rescue the effect of UBQLN2 on radiosensitization. Furthermore, by performing a co-immunoprecipitation (Co-IP) assay, we confirmed the direct interaction between UBQLN2 and p38 MAPK. These findings suggest that UBQLN2-mediated p38 MAPK activation exerts a role in the regulation of the radiosensitivity of ESCC, which may contribute to the development of radiosensitizers.

## 2. Materials and Methods

### 2.1. Cell Culture and siRNA Transfection

Human ESCC cell lines KYSE-30, KYSE-70, and KYSE-150 were purchased from Sigma-Aldrich Co. (St. Louis, USA). Ec109 cells were purchased from BnBio Inc. (Beijing, China). The normal human esophageal epithelial cell line Het-1A was bought from the American Type Culture Collection (Manassas, USA). Ec109 cells and KYSE-30 cells were maintained in the RPMI-1640 medium. KYSE-150 cells were maintained in the DMEM medium. KYSE-70 cells were maintained in MEM medium. All media were supplemented with 10% fetal bovine serum (FBS) and 1% penicillin/streptomycin. Het-1A was cultured in BEGM™ BulletKit™. Cells were kept at 37°C in a humidified 5% carbon dioxide atmosphere. For UBQLN2-siRNA transfection, UBQLN2 and negative control siRNA were obtained from Sangon Biotech (Shanghai, China). Also, the transfection was performed using Lipofectamine RNAi-Max reagent (Invitrogen, USA) in accordance with the protocol of the manufacturer.

### 2.2. Data Acquisition and Analysis

RNA-sequencing expression profiles, genetic mutations, and corresponding clinical data of patients with ESCC were downloaded from The Cancer Genome Atlas (TCGA) database (https://portal.gdc.cancer.gov) and analyzed by ACB (http://www.aclbi.com). The somatic mutation data in Mutation Annotation Format (MAF) were analyzed through the “maftools” package. The “limma” package was used to identify the difference in the UBQLN2 expression between ESCC and normal tissues. In order to evaluate the prognostic role of UBQLN2 in ESCC, a nomogram model was established. Univariate and multivariate Cox regression analyses were performed to determine the candidate items for the construction of the nomogram. The correlation between the UBQLN2 expression level and survival time of ESCC patients in the TCGA cohort and our own cohort was investigated via the Kaplan–Meier method.

### 2.3. Western Blotting

Cells were suspended in a radioimmunoprecipitation assay (RIPA) buffer containing phosphatase and protease inhibitors. BCA Protein Assay Kit (Beyotime, China) was used to analyze the concentration of protein. The same amount of protein samples were separated with 10% SDS-PAGE and relocated onto polyvinylidene difluoride membranes (Millipore, USA), followed by incubating with 5% skim milk to block the nonspecific binding of antibodies. After that, the membranes were incubated with primary antibodies against UBQLN2 (Abcam, UK), GAPDH (Bioss, China), phosphorylated DNA-dependent protein kinase catalytic subunit (p-DNA-PKcs) (Abcam, UK), DNA-PKcs (Abcam, UK), and p38 MAPK (Abcam, UK) overnight at 4°C, and then incubated with horseradish peroxidase (HRP)-conjugated goat anti-mouse/rabbit secondary antibodies (ZSGB-bio, China). Finally, the bands were visualized by an enhanced chemiluminescence detection system. GAPDH was used as the internal control.

### 2.4. Colony Formation Assay

The same amount of cells (500 cells) were seeded into a 6-well cell culture plate. After 2 weeks of culture, the plates were stained with 0.1% crystal violet (Sangon Biotech, China) and incubated at 37°C for 30 min. Then, the colonies were counted after destaining.

### 2.5. RNA-Sequencing (RNA-Seq) and Data Analysis

According to the manufacturer's specifications, RNA was extracted from Ec109 and UBQLN2 knockdown Ec109 cells using the RNeasy Micro Kit (Qiagen, USA). RNA quality was assessed with a 2100 Bioanalyzer (Agilent, USA). RNA-seq was performed by Aksomics Inc., China. Briefly, the KAPA Stranded RNA-seq Library Prep Kit (Illumina, USA) was used to build the RNA-seq library. Final libraries were sequenced on Illumina HiSeq 4000 with paired-end 2 × 150-bp read lengths. Use FastQC to control the quality of the raw reads. The reads were then aligned to the human genome (hg19) using STAR. Differential gene expression data were generated using DESeq2. Subsequent analyses were carried out using R (3.4.2).

### 2.6. Co-Immunoprecipitation (Co-IP) Assay

The interaction between proteins and proteins was analyzed by the Co-IP assay. First, 293T cells were co-transfected with flag-labeled UBQLN2 and Myc-labeled p38 MAPK. Then, the transfected cells were lysed in an IP buffer containing 20 mM Tris-HCl (pH 7.4), 150 mM NaCl, 1 mM EDTA (pH 8), 1% Nonidet P-40, 1× Protease, and Phosphatase Inhibitor Cocktail. Subsequently, the cell lysis was incubated with the indicated antibodies for 1 h at 4°C and then with protein A/G Plus-agarose (Santa Cruz Biotechnology, USA) for another 1 h at 4°C. Finally, the interaction protein was pulled down by the magnetic beads and detected by immunoblotting.

### 2.7. Animal Study

Twenty-four female BALB/c nude mice (CAS, Beijing, China) were divided into four groups according to the type of Ec109 cells inoculated. Group 1: wild-type (WT); Group 2: WT + irradiation (IR); Group 3: UBQLN2 knockdown (UBQLN2-KD) +IR; and Group 4: UBQLN2-KD + IR + SB203580. WT or UBQLN2-KD Ec109 cells were collected and suspended in 100 *μ*l of PBS at a concentration of 1 × 10^6^ cells and subcutaneously injected into mice. The tumor size was evaluated twice a week. When each tumor reached a volume of nearly 150 mm^3^, we started to irradiate them with 6Gy of X-ray and treat mice with indicated agents. After 4 weeks of follow-up, the mice were euthanized, and the subcutaneous tumors were collected for immunohistochemical analysis. The size of the tumor was calculated by the formula for volume (*V*), *V* = 0.5 × (length × width^2^). The animal study was conducted in accordance with the guidelines of the International Association for Experimental Animal Care Assessment and Certification.

### 2.8. Patients and Tissue Samples

A total of 55 patients with ESCC who underwent surgical treatment in our hospital were enrolled in this study. None of the enrolled patients received preoperative radiotherapy or chemotherapy. Formalin-fixed, paraffin-embedded tumor samples were collected from each patient. The clinicopathological characteristics of the enrolled patients are shown in Table S1. Overall survival (OS) was defined as the interval between the diagnosis of ESCC and death or the last follow-up. The research protocol was reviewed and approved by the Ethics Committee of the General Hospital of Ningxia Medical University (2019-171).

### 2.9. Immunohistochemical (IHC) Staining

IHC staining was performed on the specimens from xenograft models or ESCC patients. Antigen retrieval was carried out under high pressure. The activity of endogenous peroxidase was blocked by 3% hydrogen peroxide. After that, primary antibodies against UBQLN2 (Abcam, USA), Caspase-3 (Bioss, China), p38 MAPK (Bioss, China), and Ki-67 (Bioss, China) were diluted and incubated with tissue sections overnight at 4°C. Antigen-antibody binding was visualized with 3,3′-diaminobenzidine (DAB). Finally, the section was assessed by two pathologists and evaluated using a semiquantitative method as previously described [17].

### 2.10. Statistical Analysis

A student's *t*-test or analysis of variance (ANOVA) was used to evaluate the differences among the groups. *χ*^2^ test was used to analyze the association of categorical variables. Survival was estimated by the Kaplan–Meier method and compared by the log-rank test. *P* < 0.05 indicated a statistical significance. All statistics were performed using SPSS 22.0 for Windows (Armonk, USA) and GraphPad Prism 8.0 software (San Diego, USA). All the data displayed were representative, and all the experiments were repeated at least three times independently.

## 3. Results

### 3.1. The Prognostic Role of UBQLN2 in Esophageal Cancer

**Figure 1:**
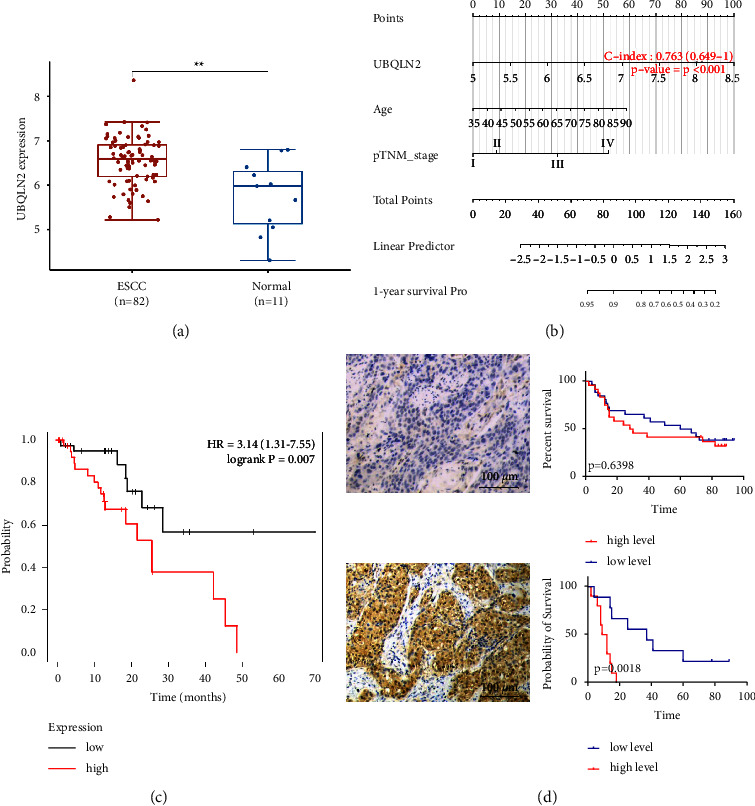
The prognostic role of UBQLN2 in patients with esophageal squamous cell carcinoma (ESCC). (a) A box and bar graph comparing the levels of UBQLN2 mRNA between ESCC and normal paracancerous tissues. ^*∗∗*^*P* < 0.01 of Student's *t*-test. (b) Nomogram model predicting the 1-year overall survival (OS) of patients with ESCC. The high expression of UBQLN2 contributed nearly 100 points, which was higher than that of age and pTNM staging. (c) Kaplan–Meier analysis of OS in ESCC patients with the different expression levels of UBQLN2 mRNA in cancer tissue. Data from The Cancer Genomic Atlas (TCGA). (d) Negative (top) and positive (bottom) immunohistochemical (IHC) staining of UBQLN2 in ESCC tissues of our own cohort. Kaplan–Meier analysis demonstrated that the expression level of UBQLN2 had no effect on the survival of patients with ESCC in our own cohort (*P* = 0.6398). However, in ESCC patients with lymph node metastasis, the OS of patients with low expression of UBQLN2 was significantly better than that of patients with high expression of UBQLN2 (*P* = 0.0018).

First, the expression level of UBQLN2 mRNA was evaluated in the TCGA database, and we found that the expression level of UBQLN2 mRNA in ESCC was higher than that in normal paracancerous tissues (Figure 1(a)). Based on the available clinical data in the TCGA database, we also established a prognostic nomogram, which integrated all significant OS prognostic indicators created in Cox regression analysis (Figure 1(b)). The C-index for OS prediction was 0.763. The high expression of UBQLN2 contributed nearly 100 points to the total points, which was higher than those contributed by the age and pTNM stage, suggesting that UBQLN2 played a more important role in the prognosis of patients with ESCC than other risk factors. Subsequently, we studied the correlation between the expression level of UBQLN2 mRNA in the TCGA database and the survival of patients with ESCC. The results showed that OS in patients with high expression of UBQLN2 was more unfavorable than that of patients with low expression of UBQLN2 (Figure 1(c)). Finally, the expression of UBQLN2 was detected by IHC in 55 ESCC patients who underwent surgery in our hospital (Table S1). The results of IHC showed that UBQLN2 was mainly located in the cytoplasm of esophageal cancer cells and also weakly stained in the nucleus. According to the results of IHC staining, 45.5% of ESCC patients showed a high expression of UBQLN2. Furthermore, Kaplan–Meier analysis demonstrated that the expression level of UBQLN2 had no effect on the survival of patients with ESCC (*P* = 0.64). However, in the subgroup of ESCC patients with lymph node metastasis, the OS of patients with low expression of UBQLN2 was significantly better than that of patients with high expression of UBQLN2 (*P* < 0.01) (Figure 1(d)). Taken together, these results confirm the prognostic role of UBQLN2 expression level in patients with ESCC.

### 3.2. Inhibition of UBQLN2 Expression Promotes the Sensitivity of Esophageal Cancer Cells to Radiotherapy

**Figure 2:**
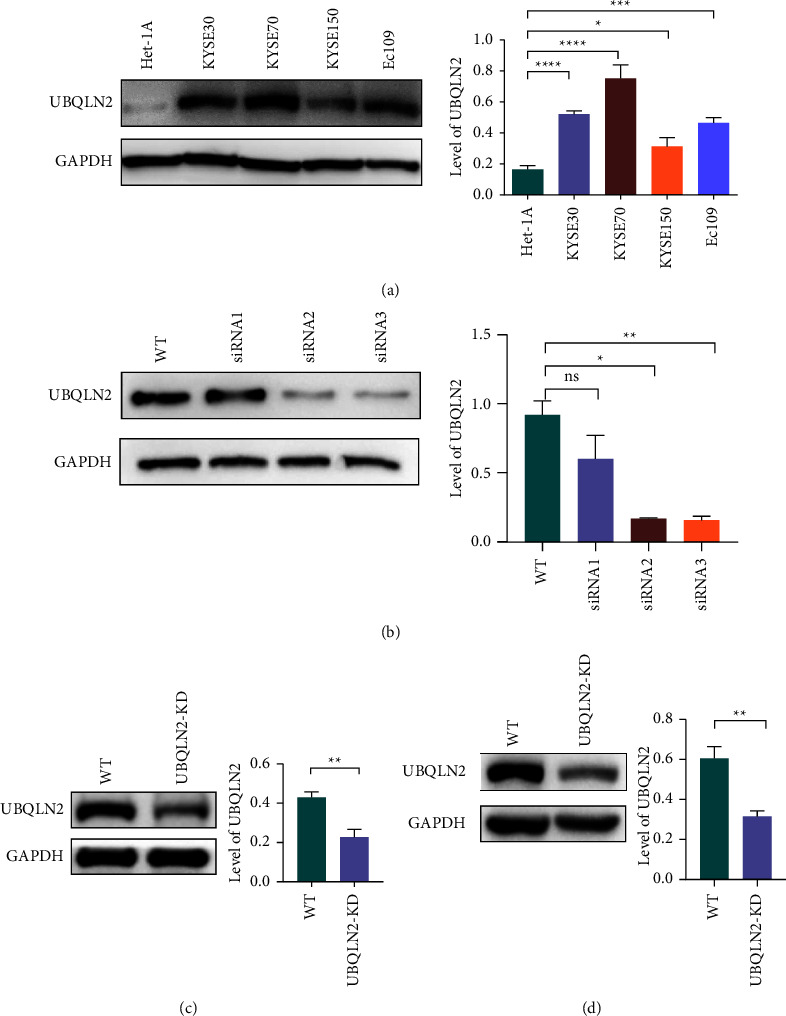
The expression level of UBQLN2 protein in esophageal squamous cell carcinoma (ESCC) cells. (a) The protein expression level of UBQLN2 in four ESCC cell lines and the normal esophageal epithelial cell line Het-1A was detected by Western blotting. (b) The expression of UBQLN2 protein in UBQLN2-siRNAs transfected Ec109 cells was detected by Western blotting. The transfection effect of UBQLN2-siRNA was confirmed by Western blotting at protein levels in Ec109 (c) and KYSE-30 (d) cells. All results are presented as the mean ± SEM from three repeated experiments. ^*∗*^*P* < 0.05, ^*∗∗*^*P* < 0.01, ^*∗∗∗*^*P* < 0.001, and ^*∗∗∗∗*^*P* < 0.0001 of the Student's *t*-test or analysis of variance (ANOVA).

To investigate the relationship between the expression of UBQLN2 and the radiosensitivity of ESCC cells, we first detected the expression of UBQLN2 in KYSE-30, KYSE-70, KYSE-150, and Ec109 ESCC cell lines and normal human esophageal epithelial cells HET-1A. Western blotting results showed that the expression of UBQLN2 in four ESCC cell lines was significantly upregulated compared with that of a normal esophageal epithelial cell line (Figure 2(a)).

**Figure 3:**
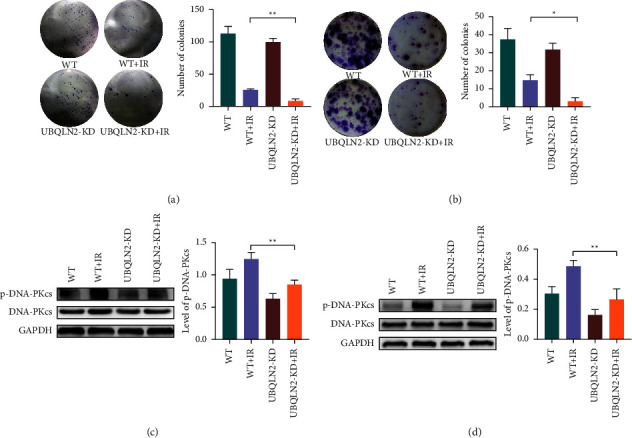
Silencing UBQLN2 enhanced the radiosensitivity of esophageal squamous cell carcinoma (ESCC) cells. The radiosensitivity of wild-type (WT) or UBQLN2 knockdown (UBQLN2-KD) Ec109 (a) and KYSE-30 (b) cells was detected by a clone formation assay. Representative pictures of the colony formation assay are given. Western blotting analysis showed that silencing UBQLN2 could significantly inhibit the protein expression of the phosphorylated DNA-dependent protein kinase catalytic subunit (p-DNA-PKcs) in Ec109 (c) and KYSE-30 (d) cells after irradiation (IR). All results are presented as the mean ± SEM from three repeated experiments. ^*∗*^*P* < 0.05 and ^*∗∗*^*P* < 0.01 of analysis of variance (ANOVA).

To further study whether UBQLN2 can mediate the radiosensitivity of ESCC cells in vitro, Ec109 and KYSE-30 cell lines with moderate UBQLN2 expression were subsequently transfected with UBQLN2-siRNA. The candidate siRNA and knockdown efficiency were verified by Western blotting (Figures 2(b)–2(d)). Then, the UBQLN2-repressed ESCC cells and the corresponding control ESCC cells were subjected to 4Gy of irradiation. A colony formation assay was used to detect cell proliferation after irradiation. The results showed that UBQLN2 knockdown could significantly inhibit the cloning ability of ESCC cells in response to irradiation (Figures 3(a) and 3(b)).

It is well known that the repair ability of DNA injuries is closely related to the intrinsic radiosensitivity of cells. In order to further confirm the effect of UBQLN2 expression levels on the radiosensitivity of ESCC cells, the protein expression levels of RPA70, XRCC-2, XRCC-4, Ligase IV, Ku70, RAD51, and p-DNA-PKcs involved in the homologous recombination (HR) and nonhomologous DNA terminal junction (NHEJ) DNA repair pathways were detected by Western blotting. We found that inhibition of UBQLN2 could significantly reduce the protein expression level of p-DNA-PKcs, which is induced by irradiation (Figures 3(c) and 3(d)). The results of other tested proteins are shown in Figure S1. These data indicated that silencing UBQLN2 could enhance the radiosensitivity of ESCC cells.

### 3.3. UBQLN2 Enhances the Radiosensitivity of ESCC Cells by Activating p38 MAPK

**Figure 4:**
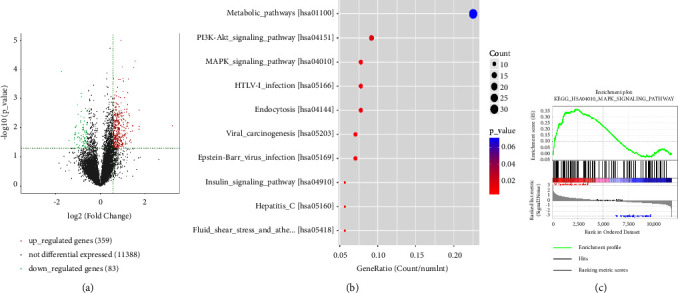
RNA-sequencing results. (a) Volcano plot displaying upregulated or downregulated genes in Ec109 cells in response to UBQLN2 knockdown. (b) Kyoto Encyclopedia of Genes and Genomes (KEGG) pathways were significantly enriched for upregulated genes in UBQLN2 knockdown Ec109 cells. (c) Gene set enrichment analysis (GSEA) analysis also identified the mitogen-activated protein kinases (MAPK) signaling pathway as a regulatory target of UBQLN2 in RNA-sequencing data.

**Figure 5:**
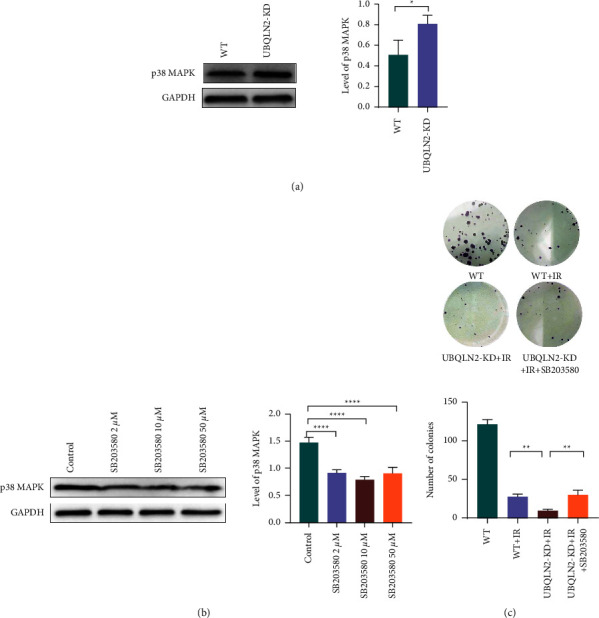
Silencing UBQLN2 enhanced the radiosensitivity of esophageal squamous cell carcinoma (ESCC) cells via activating p38 mitogen-activated protein kinases (MAPK) signaling. (a) Western blotting analysis showed that the expression of p38 MAPK in Ec109 cells was upregulated after UBQLN2 knockdown. (b) Western blotting analysis showed the efficacy of different concentrations of the p38 MAPK inhibitor SB203580 on the protein expression of p38 MAPK in Ec109 cells. (c) Colony formation assay showed that SB203580 could reverse the radiosensitization of Ec109 cells mediated by UBQLN2. All results are presented as the mean ± SEM from three repeated experiments. ^*∗*^*P* < 0.05, ^*∗∗*^*P* < 0.01, and ^*∗∗∗∗*^*P*< 0.0001 of analysis of variance (ANOVA).

Next, we explored the mechanisms of UBQLN2-modulated radiosensitivity. RNA-seq showed that there were 440 differentially expressed genes (DEGs) in UBQLN2 knockdown ESCC cells compared with the corresponding wild-type ESCC cells. Among them, 358 genes were upregulated and 82 genes were downregulated (Figure 4(a)). The Kyoto Encyclopedia of Genes and Genomes (KEGG) (Figure 4(b)) and Gene set enrichment analysis (GSEA) (Figure 4(c)) indicated that these upregulated DEGs were mainly enriched in the MAPK signaling pathway. Therefore, in order to verify the critical role of the MAPK signaling pathway in the regulation of UBQLN2-mediated radiosensitization of ESCC cells, we used Western blotting to detect the key molecules of this pathway, including extracellularly regulated protein kinases (ERK), p38 MAPK, Jun-amino-terminal kinase (JNK), and ERK5. As shown in Figure 5(a), silencing UBQLN2 in Ec109 cells obviously increased the protein level of p38 MAPK. The results of JNK and ERK5 protein expression are shown in Figure S2.

In order to further determine whether the sensitizing effect of UBQLN2 on irradiation is due to the activation of p38 MAPK, we treated UBQLN2 knockdown Ec109 cells with different concentrations of p38 MAPK inhibitor SB203580. Western blotting results showed that 10*μ*M SB203580 could significantly inhibit the expression of p38 MAPK in UBQLN2 knockdown ESCC cells (Figure 5(b)). Subsequently, rescue experiments were performed. UBQLN2 knockdown Ec109 cells were treated with 10 *μ*M SB203580, and a colony formation assay was carried out. The results showed that inhibition of p38 MAPK could reverse the radiosensitization effect induced by silencing UBQLN2 (Figure 5(c)).

**Figure 6:**
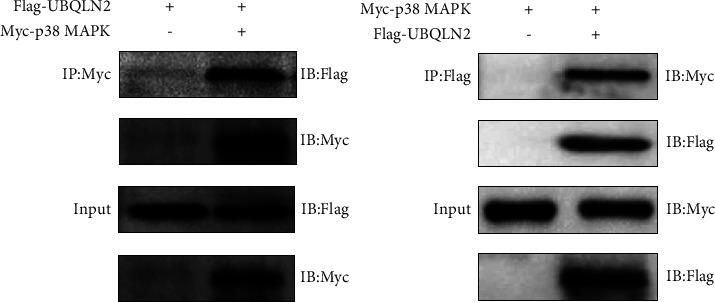
UBQLN2 directly interacted with p38 mitogen-activated protein kinases (MAPK). Exogenous co-immunoprecipitation (CO-IP) showed the interaction between UBQLN2 and p38 MAPK. The results were repeated three times.

To further confirm the relationship between UBQLN2 and p38 MAPK, a CO-IP assay was performed. The results revealed that UBQLN2 could interact with p38 MAPK in a reciprocal fashion (Figure 6).

In summary, these results suggest that UBQLN2 may affect the radiosensitivity of ESCC cells by activating p38 MAPK. UBQLN2 upregulates p38 MAPK through direct interaction.

### 3.4. Knockdown UBQLN2 Increases the Radiosensitivity of Ec109 Xenograft Tumors

**Figure 7:**
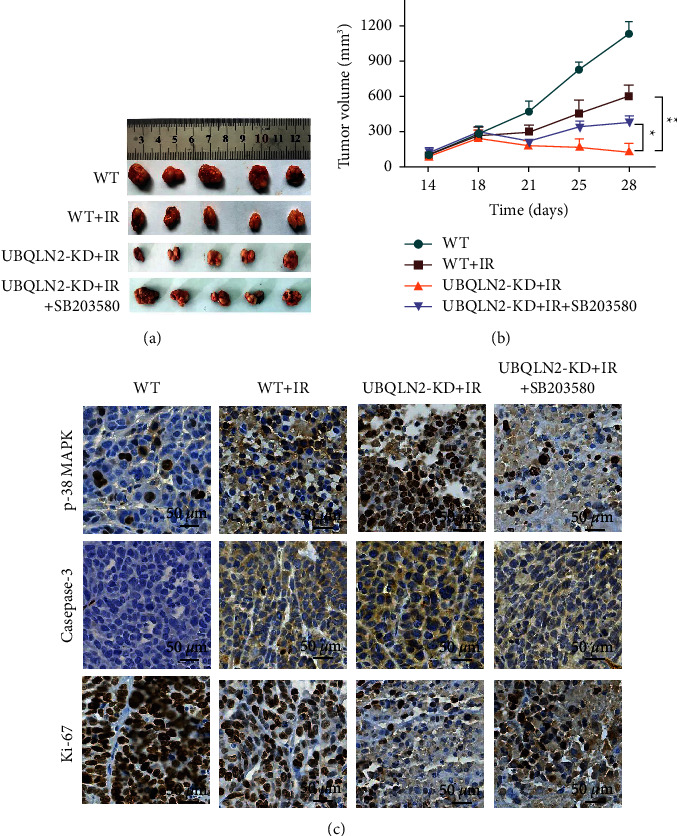
Knockdown of UBQLN2 enhanced the sensitivity of radiotherapy for esophageal squamous cell carcinoma (ESCC) xenografts. (a) The tumor volume of xenografts in the wild-type (WT), WT + irradiation (IR), UBQLN2 knockdown (UBQLN2-KD) +IR, and UBQLN2-KD + IR + SB203580 group. (b) The curves of xenograft growth overtime in WT, WT + IR, UBQLN2-KD + IR, and UBQLN2-KD + IR + SB203580 groups. (c) Immunohistochemical (IHC) analysis of p38 mitogen-activated protein kinases (MAPK), caspase-3, and Ki-67 on tissue sections of ESCC xenografts. The data are presented as the mean ± SD from six mice. ^*∗*^*P* < 0.05 and ^*∗∗*^*P* < 0.01 of analysis of variance (ANOVA).

To further determine whether UBQLN2 has the radiosensitizing effect in vivo, WT and UBQLN2-KD Ec109 cells were subcutaneously inoculated into female BALB/c nude mice. When the tumor volume reached 150 mm^3^, the tumor-bearing mice were irradiated with 6Gy. The results revealed that, after irradiation, the volume of UBQLN2 knockdown Ec109 xenograft tumors (UBQLN2-KD + IR group) was significantly smaller than that of wild-type Ec109 tumors (WT + IR group). However, a p38 MAPK inhibitor could reverse the radiosensitization effect of silencing UBQLN2 (Figures 7(a) and 7(b)). IHC analysis showed that the expression of p38 MAPK and caspase-3 in the tumor tissues of the UBQLN2-KD + IR group was significantly higher than that in the WT + IR group. In addition, the expression of Ki-67 in the tumor tissues of the UBQLN2-KD + IR group was obviously suppressed (Figure 7(c)). These observations provide further evidence for the role of UBQLN2 in regulating the radiosensitivity of ESCC.

## 4. Discussion

UBQLN2 is a factor necessary to maintain protein homeostasis and cell survival by degrading misfolded or damaged proteins, which accumulate upon stimulation with various stresses [18, 19]. Recent studies have shown that UBQLN2 may promote cell proliferation and invasion in various types of cancer. UBQLN2 had been identified as a risk factor for osteosarcoma [14] and a novel marker for detecting urothelial cancer cells [13]. Overexpression of UBQLN2 in hepatocellular carcinoma patients indicates a poor prognosis [10]. However, loss of UBQLN2 led to an epithelial-to-mesenchymal transition (EMT)-like change in lung adenocarcinoma cells and promoted the invasion and metastasis of lung cancer, as was also demonstrated [11, 12, 15].

**Figure 8:**
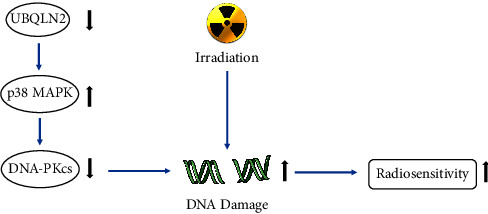
Schematic presentation of UBQLN2 modulation of radiosensitivity in esophageal squamous carcinoma (ESCC).

In the present study, we first explored the prognostic role of UBQLN2 in patients with ESCC. We found that the UBQLN2 expression level was correlated with prognosis in ESCC patients, and longer OS in ESCC patients with low UBQLN2 expression than those with a high UBQLN2 expression. Our results are consistent with the findings in other types of cancer. Next, we evaluated the role of UBQLN2 in modulating the radiosensitivity of ESCC cells. Our data revealed that silencing UBQLN2 increased the radiosensitivity of ESCC cells. Further supporting this observation, RNA-seq analysis was performed. We identified p38 MAPK expression as being significantly upregulated in ESCC cells upon UBQLN2 suppression. P38 MAPK signaling plays a variety of roles in the cell process, depending on cell type and stimulation, and has been shown to promote not only cell death but also cell growth and survival [20, 21]. A series of proinflammatory cytokines and environmental stress can trigger the p38 MAPK cascade [22]. P38 MAPK signaling is also thought to affect the radiosensitivity of cancer cells because its activity is associated with G2 cell cycle checkpoint control [23]. Inhibition of p38 MAPK by SB203580 or overexpression of a dominant-negative form of p38 effectively blocked the activation of caspases after irradiation [24]. In the current study, our findings suggest that UBQLN2 knockdown may increase the radiosensitivity of ESCC cells exposed to X-ray irradiation through activating the p38 MAPK signaling pathway. Inhibiting the expression of p38 MAPK can reduce the radiosensitivity of UBQLN2 knockdown ESCC cells to X-ray irradiation. In the further mechanism study, we found that UBQLN2 mediated the expression of p38 MAPK through direct interaction with p38 MAPK. These results suggest that UBQLN2 may affect the radiosensitivity of ESCC cells by activating p38 MAPK. Apart from p38 MAPK activation, we have clearly demonstrated that UBQLN2 is also involved in radiation-induced DNA damage repair. We found that the level of activated DNA-PKcs, an important protein involved in NHEJ repair [25], was substantially decreased in ESCC cell lines with UBQLN2 suppression, which further confirmed that downregulation of UBQLN2 impeded DNA damage NHEJ repair and increased the radiosensitivity of ESCC cells (Figure 8). Furthermore, our in vivo data correspondingly showed that the suppression of UBQLN2 significantly improved the response to irradiation in treated animals. Taken together, these results reveal that modulation of UBQLN2 expression could hold great promise as part of a novel therapeutic strategy to improve the efficacy of radiotherapy for ESCC patients.

## 5. Conclusions

In summary, in the present study, we have found that UBQLN2 exerts a critical role in the mediation of radiosensitivity in ESCC cells. We have provided new insights into the molecular mechanism for UBQLN2 actions in radiosensitivity, which mediates DNA-PKcs to block the NHEJ repair of DNA damages induced by irradiation and activating the downstream molecule p38 MAPK, which might have a great implication in developing more effective cancer radiotherapy.

## Data Availability

All the remaining data are available within the article or from the corresponding author upon request.

## Abbreviations

ALS: Amyotrophic lateral sclerosis
ANOVA: Analysis of variance
CO-IP: Co-immunoprecipitation
DEGs: Differentially expressed genes
EMT: Epithelial-to-mesenchymal transition
ERK: Extracellular regulated protein kinases
ESCC: Esophageal squamous cell carcinoma
FBS: Fetal bovine serum
GSEA: Gene set enrichment analysis
HR: Homologous recombination
IHC: Immunohistochemical
IR: Irradiation
JNK: Jun-amino-terminal kinase
KEGG: Kyoto Encyclopedia of Genes and Genomes
MAF: Mutation Annotation Format
MAPK: Mitogen-activated protein kinases
NHEJ: Nonhomologous DNA terminal junction
OS: Overall survival
p-DNA-PKcs: Phosphorylated DNA-dependent protein kinase catalytic subunits
RIPA: Radioimmunoprecipitation assay
TCGA: The Cancer Genomic Atlas
UBA: Ubiquitin-associated domain
UBL: Ubiquitin-like domain
UBQLN2: Ubiquilin 2
WT: Wild-type.

## Supplementary Materials

Supplementary Materials Supplementary figures: Supplementary Figure 1. Western blotting analysis of RPA70, XRCC-2, XRCC-4, Ligase IV, Ku70, and RAD51 proteins in Ec109 and KYSE-30 cell lines in wild-type (WT) group, WT+ irradiation (IR) group, Ubiquilin 2 knockdown (UBQLN-KD) group and UBQLN-KD+IR group. Supplementary Figure 2. Western blotting analysis of Jun-amino-terminal kinase (JNK) and extracellular regulated protein kinases 5 (ERK5) proteins in Ec109 and KYSE-30 cell lines i2n wild-type (WT) group, WT+ irradiation (IR) group, Ubiquilin 2 knockdown (UBQLN-KD) group, and UBQLN-KD+IR group. Supplementary table: Supplementary Table 1. Clinicopathological characteristics of esophageal squamous cell carcinoma (ESCC) patients with different UBQLN2 expression levels.
