# Supplementary material for: Silencing UBQLN2 Enhances the Radiosensitivity of Esophageal Squamous Cell Carcinoma (ESCC) via Activating p38 MAPK
Source: J Oncol. 2023 Jan 5;2023:2339732. doi: 10.1155/2023/2339732 (PMC9836790; doi:10.1155/2023/2339732)
Supplement: Supplementary Materials — Supplementary figures: Supplementary Figure 1. Western blotting analysis of RPA70, XRCC-2, XRCC-4, Ligase IV, Ku70, and RAD51 proteins in Ec109 and KYSE-30 cell lines in wild-type (WT) group, WT+ irradiation (IR) group, Ubiquilin 2 knockdown (UBQLN-KD) group and UBQLN-KD+IR group. Supplementary Figure 2. Western blotting analysis of Jun-amino-terminal kinase (JNK) and extracellular regulated protein kinases 5 (ERK5) proteins in Ec109 and KYSE-30 cell lines i2n wild-type (WT) group, WT+ irradiation (IR) group, Ubiquilin 2 knockdown (UBQLN-KD) group, and UBQLN-KD+IR group. Supplementary table: Supplementary Table 1. Clinicopathological characteristics of esophageal squamous cell carcinoma (ESCC) patients with different UBQLN2 expression levels. [file 2339732.f1.zip › Table S1.docx]

Table S1 Clinicopathological characteristics of esophageal squamous cell carcinoma (ESCC) patients with different UBQLN2 expression level.

| Clinicopathologic characteristics | No. of patients | High UBQLN2 level | Low UBQLN2 level | P value |
| --- | --- | --- | --- | --- |
|  |  |  |  |  |
| Gender |  |  |  |  |
| Male | 47 | 23 | 24 |  |
| Female | 8 | 2 | 6 | 0.209 |
| Age (years) |  |  |  |  |
| <60 | 16 | 8 | 8 |  |
| ≥60 | 39 | 17 | 22 | 0.665 |
| KPS |  |  |  |  |
| >80 | 35 | 19 | 16 |  |
| ≤80 | 20 | 6 | 14 | 0.082 |
| Weight loss (kg) |  |  |  |  |
| <5 | 42 | 17 | 25 |  |
| ≥5 | 13 | 8 | 5 | 0.183 |
| Drinking |  |  |  |  |
| Yes | 17 | 10 | 7 |  |
| No | 38 | 15 | 23 | 0.184 |
| Smoking |  |  |  |  |
| Yes | 32 | 18 | 14 |  |
| No | 23 | 7 | 16 | 0.058 |
| Histological differentiation |  |  |  |  |
| Well and moderate | 33 | 16 | 17 |  |
| Poor | 22 | 9 | 13 | 0.580 |
| Tumor length (cm) |  |  |  |  |
| <3 | 12 | 5 | 7 |  |
| ≥3 | 43 | 20 | 23 | 0.766 |
| Infiltration depth |  |  |  |  |
| T1 and T2 | 20 | 9 | 11 |  |
| T3 and T4 | 35 | 16 | 19 | 0.959 |
| Lymph node status |  |  |  |  |
| N0 | 35 | 15 | 20 |  |
| N1 | 20 | 10 | 10 | 0.609 |
| pTNM staging |  |  |  |  |
| I and II | 38 | 17 | 21 |  |
| III | 17 | 8 | 9 | 0.873 |

KPS: Karnofsky; TNM: tumor, node and metastasis.
