# Supplementary figures and images for: Silencing UBQLN2 Enhances the Radiosensitivity of Esophageal Squamous Cell Carcinoma (ESCC) via Activating p38 MAPK
Source: J Oncol. 2023 Jan 5;2023:2339732. doi: 10.1155/2023/2339732 (PMC9836790; doi:10.1155/2023/2339732)

## Ec109

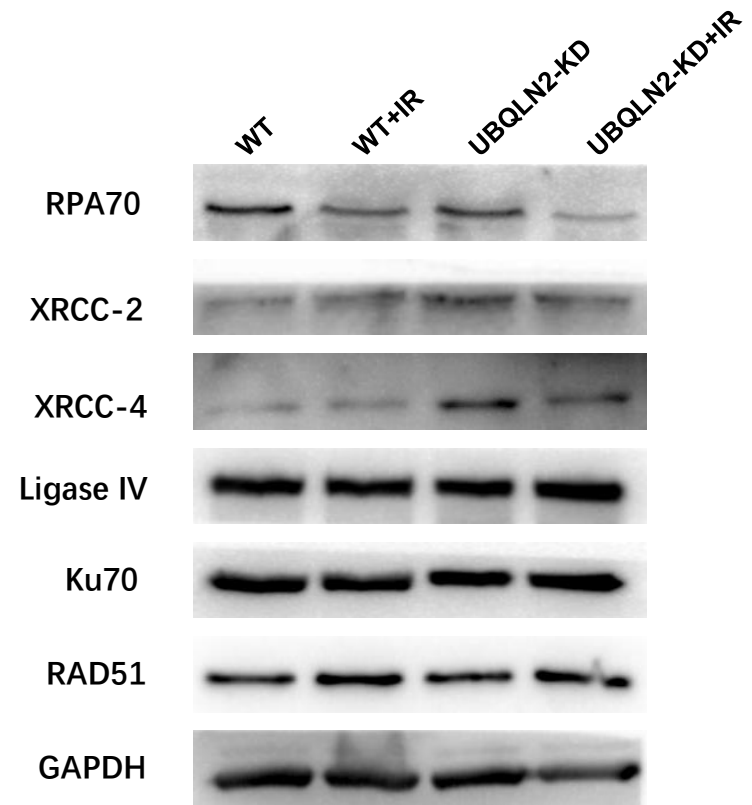

## KYSE-30

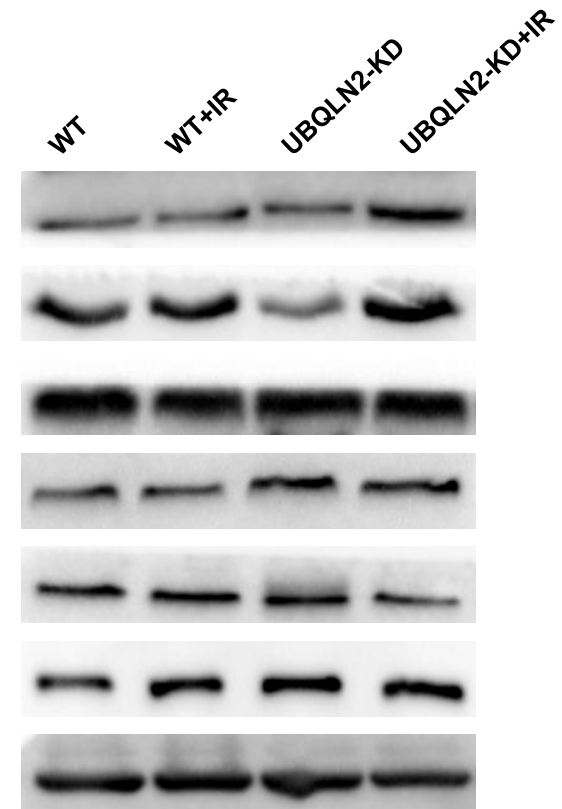

Supplement: Supplementary Materials — Supplementary figures: Supplementary Figure 1. Western blotting analysis of RPA70, XRCC-2, XRCC-4, Ligase IV, Ku70, and RAD51 proteins in Ec109 and KYSE-30 cell lines in wild-type (WT) group, WT+ irradiation (IR) group, Ubiquilin 2 knockdown (UBQLN-KD) group and UBQLN-KD+IR group. Supplementary Figure 2. Western blotting analysis of Jun-amino-terminal kinase (JNK) and extracellular regulated protein kinases 5 (ERK5) proteins in Ec109 and KYSE-30 cell lines i2n wild-type (WT) group, WT+ irradiation (IR) group, Ubiquilin 2 knockdown (UBQLN-KD) group, and UBQLN-KD+IR group. Supplementary table: Supplementary Table 1. Clinicopathological characteristics of esophageal squamous cell carcinoma (ESCC) patients with different UBQLN2 expression levels. [file 2339732.f1.zip › figure s1.pdf]

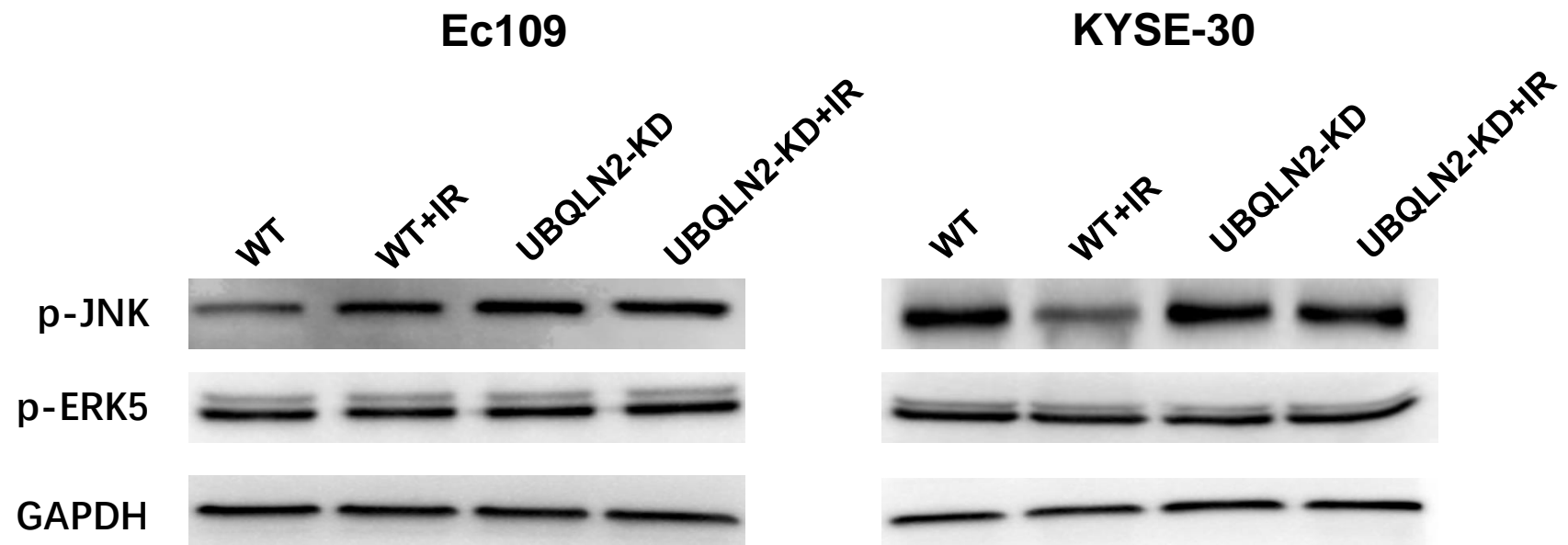

Supplement: Supplementary Materials — Supplementary figures: Supplementary Figure 1. Western blotting analysis of RPA70, XRCC-2, XRCC-4, Ligase IV, Ku70, and RAD51 proteins in Ec109 and KYSE-30 cell lines in wild-type (WT) group, WT+ irradiation (IR) group, Ubiquilin 2 knockdown (UBQLN-KD) group and UBQLN-KD+IR group. Supplementary Figure 2. Western blotting analysis of Jun-amino-terminal kinase (JNK) and extracellular regulated protein kinases 5 (ERK5) proteins in Ec109 and KYSE-30 cell lines i2n wild-type (WT) group, WT+ irradiation (IR) group, Ubiquilin 2 knockdown (UBQLN-KD) group, and UBQLN-KD+IR group. Supplementary table: Supplementary Table 1. Clinicopathological characteristics of esophageal squamous cell carcinoma (ESCC) patients with different UBQLN2 expression levels. [file 2339732.f1.zip › figure s2.pdf]
